# Restorative Effects of Biophilic Workplace and Nature Exposure during Working Time: A Systematic Review

**DOI:** 10.3390/ijerph20216986

**Published:** 2023-10-27

**Authors:** Gabriela Gonçalves, Cátia Sousa, Maria Jacinta Fernandes, Nuno Almeida, António Sousa

**Affiliations:** 1 Department of Psychology and Educational Sciences, Faculty of Human and Social Sciences, University of Algarve, Campus de Gambelas, 8005-139 Faro, Portugal; nmfalmeida@ualg.pt; 2 Centre for Research in Psychology (CIP/UAL), University of Algarve, 8005-139 Faro, Portugal; 3 Higher School of Management, Hospitality and Tourism, University of Algarve, Campus da Penha, 8005-139 Faro, Portugal; 4 Faculty of Sciences and Technology, University of Algarve, Campus de Gambelas, 8005-139 Faro, Portugal; mfernan@ualg.pt; 5 Department of Mechanical Engineering, Higher Institute of Engineering, University of Algarve, Campus da Penha, 8005-139 Faro, Portugal; asousa@ualg.pt

**Keywords:** systematic review, workplace, restorative effect, organizational behaviour, well-being, motivation, satisfaction, work performance

## Abstract

The work environment plays a crucial role in the health and performance of employees. The growing interest in workers’ well-being has driven the inclusion of nature in workplaces, despite many employees spending most of their time indoors, away from nature. Studies show that biophilic design in offices can have positive effects and promote workers’ well-being. However, research on the beneficial effects of nature exposure in the workplace is limited and scattered. Thus, the aim of this systematic review was to consolidate current knowledge on the restorative effects of nature exposure on workers during work activities. Different types of exposure, both outdoors and indoors, were considered, with a focus on outcomes related to well-being, motivation, job satisfaction, and work performance. Out of the initially identified 1225 articles, only 16 met the criteria for analysis. Although the analysed studies provided compelling evidence regarding the restorative effects of nature exposure in the workplace, the review also points out gaps and limitations concerning the number of specific studies in this area and the need to adequately assess the sensory dimensions involved in these effects. Conducting more comprehensive and multidimensional investigations into the impacts of nature on the work environment could contribute to guiding more effective design strategies and creating healthier and more productive workplaces for employees.

## 1. Introduction

Human behaviour entails considering the individual, their attributes, sensory traits, and the significance of their surroundings, while also considering the environment in terms of its physical and social elements, such as dimensions, colours, sounds, and people. This interaction can lead to different effects on individuals. Thus, some environments and environmental components can be sources of stress, while others have the potential to promote recovery from it [1,2,3]. This recovery is particularly relevant given the demands individuals face daily, especially in a work context.

Restorative environments, as defined by von Lindern et al. (2017), are those that exert little pressure on the individual’s physiological and psychological resources. These environments allow for the restoration of cognitive–emotional and functional resources and capacities that may have been compromised by stress or daily demands [4,5,6]. Attention restoration theory [7] and stress recovery theory [8] support the notion that safe natural environments are far more restorative than non-natural ones [9].

Natural environments can capture an individual’s involuntary attention, facilitating the recovery of mental fatigue, attentional resources, and cognitive capacities, as well as boosting affective–emotional benefits (e.g., [10,11,12]). Immersion in natural environments is associated with a lower cognitive processing load [13] and is perceived as more restorative than urban environments [14]. Moreover, exposure to nature is linked to lower levels of psychological stress and psychophysiological stress recovery, thereby promoting well-being [9]. Natural environments have positive impacts on blood pressure regulation, heart rate, cortisol levels, and mood states (e.g., [15,16,17]). This restorative capacity of natural environments is also observable in urban natural settings, such as city gardens and parks. Several studies show that these green spaces are strongly associated with the physical and mental well-being of the urban population (e.g., [9,18,19,20,21]) and that their frequency allows for the restoration of mental capacities and stress reduction, with these effects being more pronounced in people residing in urban areas [1]. The specific characteristics of green spaces, such as the degree of naturalness and openness, and the presence of trees and water [3,22], to which some workers have access in the workplace, can provide them with significant restorative benefits.

Therefore, given that the majority of people spend a significant proportion of their time in the work environment [23], it is essential to recognize that the environment plays a crucial role in the health and performance of workers. Studies on the quality of the work environment highlight those environmental factors, such as noise, that can cause fatigue and negatively impact well-being, performance, productivity, and job satisfaction (e.g., [24,25]). The evidence presented in the literature, combined with the growing interest in the well-being and health of workers, has driven the inclusion of nature in workspaces. While some professions involve direct interaction with natural environments, such as gardeners and nature guides, this doesn’t represent the prevailing norm in the world of work. In fact, many workers spend most of their time and professional life indoors within buildings [26], away from nature. For most of them, the possibility of being exposed to nature during work hours depends, on the one hand, on the frequency of outdoor green breaks and, on the other hand, on the design of the workspace itself, which may include elements such as indoor plants, the presence of water, windows with a view of nature, and representations of landscapes (paintings, photographs, and videos) or natural sounds. According to Klotz and Bolino’s theoretical model (2021), indoor workers can experience nature during work in four main ways: (i) outdoor green breaks during the workday; (ii) incorporation of natural elements in the workspace; (iii) windows with a view of nature; and (iv) representations of nature in the indoor space, such as artificial plants, images, or videos of nature [24].

In other words, biophilic design offers opportunities to increase exposure to nature during work, and aspects such as maximizing natural lighting in offices and including components like water and plants can benefit workers’ cognitive, emotional, and prosocial resources [24]. In this context, several studies have pointed to the potential positive effects of biophilic design on urban office workers, such as its association with perceived social benefits and increased job satisfaction [27] as well as the restoration of well-being [23]. Furthermore, empirical and experimental investigations provide evidence that biophilic design in offices is associated with the perception of more pleasant work environments as well as improvements in workers’ health, well-being, productivity, and performance (e.g., [28,29,30]). According to a narrative review conducted by Korpela et al. (2015), studies show that exposure to nature during work breaks is associated with a lower perception of stress and better perceived health, among other favourable effects. This exposure can be direct or indirect, accidental or intentional, and refers to the frequency and duration of visits to indoor green spaces (e.g., indoor gardens) or nearby outdoor spaces close to the workplace, exposure to posters, images, or sounds of nature, and the number of windows with a view of nature or indoor plants present in an office [31]. Other more recent evidence also shows that nature-related work breaks, the presence of natural elements in the workplace, and the quality of the workplace’s sound and landscape environment can benefit the physical and mental health of workers, facilitate the restoration of mental capacities and resources, and alleviate fatigue, thus promoting well-being and work performance (e.g., [23,32,33,34]). For example, a narrative review of the literature indicated that “green breaks”—real or virtual immersions in natural environments—during the COVID-19 pandemic were one of the strategies adopted by people to cope with the adverse effects of working remotely at home during lockdown, although the effects on stress relief and increased cognitive task performance were inconclusive [35].

Empirical research on the beneficial effects of exposure to nature in the workplace context is still relatively limited and scattered, focusing on some types of exposure as mentioned in previous study examples. Additionally, some available studies use non-representative samples of participants, such as students or other non-working volunteers, and/or are conducted in simulated work scenarios (e.g., [34,36,37,38]). To date, there has been no systematic review that consolidates the findings from empirical research on the effects of exposure to nature in work contexts for workers and organizations. Despite various studies examining the effects of restorative environments, they are often grouped together indiscriminately. However, it is of paramount importance to assess the restorative effects more realistically, which entails conducting field studies involving workers in their everyday settings. Students and workers have distinct characteristics. It is important to note that virtual reality and other visual experiences are not the same. Therefore, the objective of this study is to conduct a systematic review that organizes the current state of knowledge on the restorative effects of nature exposure on workers during work activities. This review will address original empirical research published in peer-reviewed journals that investigates outcomes related to organizational behaviour and workers, including well-being, motivation, job satisfaction, and work performance. All types of nature exposure during work will be considered, including outdoor breaks and exposure in indoor environments, both real and simulated. However, studies conducted in contexts unrelated to organizational work, such as extracurricular activities or remote work, will not be included in this review. To ensure the relevance of the results to the workers’ work context, the focus will be solely on studies involving samples of workers, excluding those with non-working participants, such as students.

## 2. Methods

This systematic review was conducted according to the Preferred Reporting Items for Systematic Reviews and Meta-Analyses (PRISMA) checklist and statement [39].

### 2.1. Research Strategy and Study Identification

Prior to conducting the systematic review, a review panel consisting of experts in the theoretical and methodological areas of the topic was formed, and they defined the selection criteria and variables for analysis. Through an interactive process of definition, clarification, and refinement [40,41], an internal protocol (unpublished) was generated, specifying the search strategy, selection criteria, data extraction procedures, method of quality assessment, and terms under which study data would be pooled for the systematic review.

The search terms for this review were initially developed based on the PICO statement (e.g., [42]) and the relevant literature, and were subsequently tested, refined, and finalized. As the PICO structure was originally developed for evaluating systematic reviews and meta-analyses in medical sciences, it was necessary to adapt it for this systematic review. The final search included terms that reflect the elements of the PICO structure: Population (P), Intervention or exposure (I), Comparison intervention or exposure (C), if relevant, and Outcome (O).

**Figure 1:**
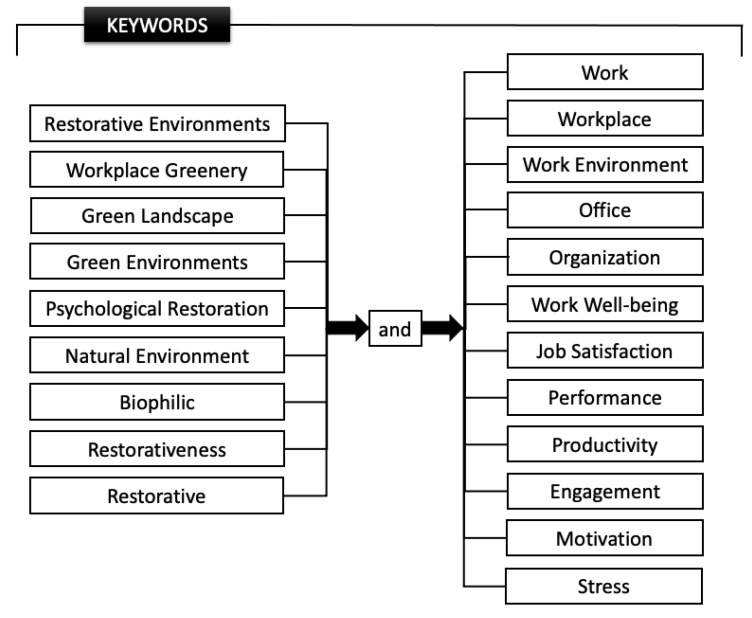
Keywords and combinations using the logical operator “and”.

Since it is an exploratory descriptive investigation, the following keywords were defined (Figure 1): “Restorative Environments”, “Workplace Greenery”, “Green Landscape”, “Green Environments”, “Psychological Restoration”, and “Natural Environment”. These keywords, due to their specificity, dimensions, and correlations, were combined using the logical operator “and” with the following terms: “work”, “workplace”, “work environment”, “office”, “organization”, “work well-being”, “job satisfaction”, “performance”, “productivity”, “engagement”, “motivation”, and “stress”.

The keywords were searched using the Title (TI) criteria, for the period from January 2000 to April 2022 (chronological parameter). The reason for selecting this period is that despite the researchers being concerned with the restorative capacity of environments starting in the 1980s [7,43], research was centred on natural environments, namely on landscapes not interfered with by humans. From the beginning of the 21st century, companies and researchers began to take an interest in the positive effects that natural elements can have on performance and well-being at work. This survey was carried out between February and July 2022 by one of the authors (NA). Subsequently, a comprehensive literature search was carried out according to the inclusion criteria, with a view to identifying all relevant investigations [41]. A total of five databases were used: Academic Search Complete, MedLine, Psychology and Behavioral Sciences Collection, PsycINFO, and Web of Science.

### 2.2. Eligibility Criteria

Articles were considered for inclusion in the review if: (a) they related to original empirical research; (b) they had been published in peer-reviewed journals; (c) they related to work contexts and environments; (d) the outcomes related to organizational behaviour; and (e) they related to working subjects. The following were excluded: (a) articles and theoretical studies; (b) opinion articles; (c) studies published in chapters and books; (d) articles in languages other than English; (e) studies related to contexts other than work and teleworking environments; and (f) samples of non-working participants (e.g., students). The application of these criteria resulted in 1225 articles that were included in the next step.

### 2.3. Conducting Searches

Of the 1225 articles obtained through the criteria, 538 repeated articles (NA; AS) were identified, reducing the total to 687 articles. After removing duplicate articles, the eligibility of the studies was assessed through a five-step process: (1) article titles; (2) screening based on abstracts; (3) full-text reading; (4) review of the full text, and (5) use of the snowball technique (e.g., [44]), which was carried out by screening the reference lists of articles resulting from step 4.

**Figure 2:**
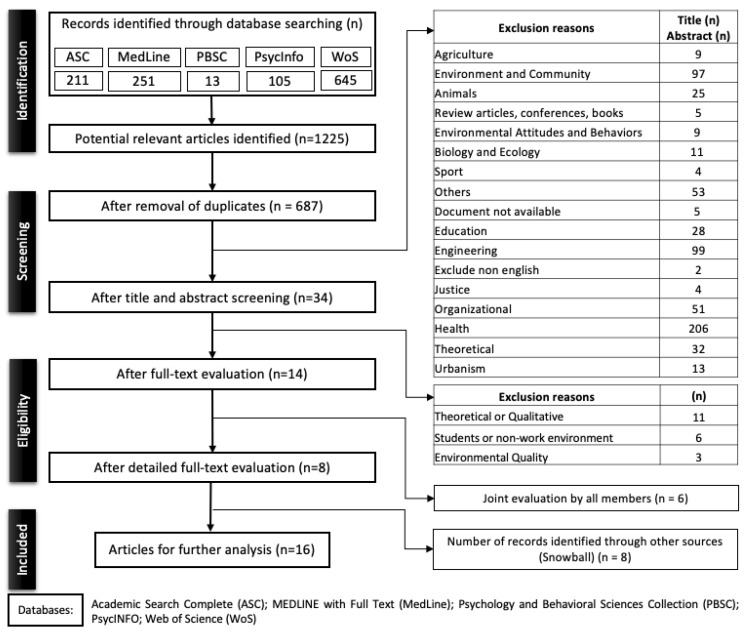
PRISMA flow diagram showing the study selection process.

Equally distributed by the authors of this study (NA; GG; MJF; AS; and CS), the articles were analysed by their title and abstract. Because of the articles that raised doubts as to their subject, a floating reading was carried out and discussed among all the authors with a view to articles being excluded or moving on to the next stage. Many of the abstracts were excluded because they did not meet the inclusion criteria (Table 1). The excluded articles were categorized according to the object of their study. Thirty-four articles progressed to the next phase for analysis of the full text, including articles that raised doubts and on which no consensus had been reached. Full-text analysis of all articles was performed by the five team members (NA; GG; MJF; AS; and CS) independently. Articles that raised doubts were evaluated jointly by all members. After reading the articles, eight progressed to the third phase (Figure 2). In the fifth stage, the snowball technique was applied to these 8 articles, which allowed 8 more articles to be chosen for analysis, resulting in a total of 16 studies for inclusion and synthesis.

### 2.4. Quality Appraisal

The 16 resulting articles from the previous stage were independently and thoroughly evaluated by two team members (CS and GG) using a checklist developed by Downs and Black (1998). The reviewers’ results were compared by an external reviewer (MJF), and any discrepancies were resolved through consensus. This checklist is a specific standard guideline for evaluation and consists of 27 items grouped into 5 dimensions of assessment, related to the reporting of results, external validity, bias, confounding factors, and study power [45]. The presence of a criterion is evaluated with the value “1”, and its absence is assigned the value “0”. When information was insufficient or not reported, “NR” (not reported) was indicated, and when a criterion was not applicable to a particular study, “NA” (not applicable) was indicated. According to Hooper et al. (2008), the quality of the articles can be assessed at different levels: excellent (26–28), good (20–25), fair (15–19), and poor (<14) [46]. The total number of points for each article was used to determine the overall quality score according to other studies (e.g., [47,48]). Given the nature of the study, we felt that some of the items were not applicable, so the values were adjusted, meaning the quality levels were weighted by the number of items considered in each article. Most articles (n = 7) were rated as being of excellent quality, and the remaining (n = 9) were rated as being of good quality.

### 2.5. Data Extraction

We defined a set of descriptive variables to characterize each of the 16 eligible studies for review: authors, year of publication, main objective, study design, sample size, type of natural elements, and restorative outcomes (Table 2). Data extraction was completed in Excel by one team member (CS) and verified by another (GG). Discrepancies were resolved by a third team member (MJF). Finally, we conducted a qualitative analysis of the results from the 16 articles. We focused on the restorative characteristics of the work environments and the observed effects on performance, well-being, and organizational behaviour variables.

## 3. Results

Out of the 16 identified studies, 9 used simulated nature, such as natural soundscapes (e.g., bird songs, water fountains, etc.) and/or visual stimuli (e.g., images of natural landscapes or plants). The remaining studies considered factors such as the type of window view and frequency of looking out of the window, access to non-simulated nature and sunlight, access to outdoor spaces, and the frequency of outdoor activities. Studies focusing on outdoor environments also consider auditory aspects, as individuals are exposed to surrounding sounds. Notably, Ma and Shu’s study (2018) investigated the different types of sounds participants were subjected to. The results showed that participants’ irritation and fatigue were significantly improved after exposure to the sounds of running water and birdsong, rather than footsteps, traffic noise, and the noise of air conditioning [33]. Similarly, Aristizabal et al. (2021) observed that immersive interventions, including nature-inspired sounds and visuals incorporating indoor plants and vegetation projections, had a positive impact. In other words, a multisensory approach reduces stress, increases satisfaction, and enhances perceptions of productivity [49].

With regard to the study design, except for Lottrup et al.’s case study (2012), all others are quantitative studies, including correlational and experimental designs (e.g., pre-post design, prospective cohort design, research intervention).

All 16 articles showed statistically significant positive relationships between exposure to nature and the restorative outcomes under study. As regards the outcomes, most identified articles focused on physiological, psychological, and organizational outcomes. In regard to physiological outcomes, most studies reported positive results, demonstrating the benefits of green and biophilic spaces. For example, the results of Yin et al.’s study (2020) showed that participants in biophilic indoor environments had consistently better recovery responses after the stressor than those in a non-biophilic environment, in terms of stress and anxiety reduction, with effects on immediate physiological responses following exposure to biophilic environments. Yin et al.’s study (2020) also observed similar results, particularly concerning physiological stress reaction indicators and creativity. In terms of psychological outcomes, several studies concluded that the relationship with nature positively impacts employees’ health and well-being (e.g., [29,53,55]). The same was observed in organizational outcomes, which highlighted job satisfaction, productivity, engagement, and organizational commitment as the most studied variables, all of which benefit from the presence of green elements in the workplace. For example, An et al.’s study (2016) showed that higher levels of exposure to natural elements were associated with greater job satisfaction and organizational commitment. Similar results were found in Shin’s study (2017), where employees with a view of the forest from their windows reported greater job satisfaction and consequently less job stress. Despite the positive effects of natural environments on organizational outcomes, Lottrup et al. (2012) observed only some significant relationships between the investigated aspects of the outdoor work environment and employee health, job satisfaction, and job performance. This may be due to most participants in the study being exposed to a green environment during their working day, even if they do not leave their workstations, as they can still see the external green environment through the windows. It is worth noting that Pasini et al.’s study (2021) was unable to assess changes in job satisfaction and engagement, as these variables were only evaluated after the experience.

In addition to the studied variables, we highlight the studies by Yeom (2021) and Lei et al. (2021) that aimed to identify the ideal amount of greenery. For example, Yeom et al. (2021) concluded that visualizing an entire green wall increases negative affect, which may be associated with confusion and a sense of confinement. This study also showed that a visual element of the green wall affects emotional impact and task load but has no effect on task performance. Lei et al. (2021) found that a 12% green coverage ratio is the ideal dose of green coverage, positively impacting both psychological and physiological outcomes, as well as productivity. The study further observed that only green coverage rates of 12% and 20% could lead to positive changes in brain physiological activities.

Studies focusing on window views in the workplace revealed positive results on the studied outcomes. For example, Shin’s study (2007) showed a significant direct effect of forest views from windows on job satisfaction and stress. Pati et al. (2008) concluded that the duration of window view observation is the second-most influential factor affecting alertness and acute stress in a sample of nurses. Among all nurses whose alertness remained the same or improved, almost 60% had exposure to the exterior and nature. In contrast, among all nurses whose alertness deteriorated, 67% were exposed to either no view or only a non-natural view. With regard to exposure to nature and sunlight, An et al. (2016) found that direct sunlight emerged as a dominant predictor of anxiety, while indirect sunlight was a dominant predictor of depressed mood, job satisfaction, and organizational commitment. On the other hand, natural elements attenuate the relationship between stressors and the studied outcome variables. Supporting similar findings, Lottrup et al. (2012, 2013) demonstrated the benefits of exposure to a green and natural outdoor environment during the workday as well as the visualization of the external environment through the office windows.

## 4. Discussion

This systematic review aimed to analyse the results of studies evaluating the restorative effects of exposure to nature on workers during their working hours, specifically focusing on outcomes related to performance, well-being, satisfaction, motivation, productivity, engagement, etc. Among the 1225 articles identified in the search, only 16 met the proposed criteria for analysis. Although the literature on this topic is starting to proliferate, it was observed during the analysis process that many articles (n = 206) evaluated only variables related to health (e.g., clinical trials, addictions, mental health, stress, dental implants, and biological effects of physical activity), while others focused on engineering areas (QAI parameters, energy performance, processes, green products, materials, corrosion, and chemistry), and yet others, although mentioning work performance, used either student samples or evaluated activities outside the work context (e.g., outdoor walking, commuting, outdoor activities). Additionally, other studies, while related to the organizational context, focused on aspects such as innovation, information technologies, and management commitment, without evaluating the impact of restorative and/or biophilic environments on employee performance. Thus, it is evident that few studies delve into this objective. This scarcity of specific studies may indicate that this area of research is still being developed, leaving room for further investigations. Studying the restorative effects of biophilic workplace environments and exposure to nature during work is important and innovative for several reasons. Firstly, the presence of natural elements in the workplace, such as plants and natural light, has been shown to enhance employee well-being, reduce stress, and promote mental health. Moreover, exposure to nature can boost employee productivity by stimulating creativity, focus, and efficiency, potentially leading to a reduction in employee absenteeism. Another significant aspect is the promotion of sustainability. Biophilic design encourages sustainable practices and environmental conservation, which is crucial in today’s world. Furthermore, companies adopting these practices may more easily attract talent by demonstrating a commitment to employee well-being and environmental responsibility. Research in this field is continuously evolving, influencing office design and architecture to create more pleasant and productive workspaces. Therefore, the uniqueness of this study stands out due to its integrated approach, prioritizing employee well-being, incorporating natural elements into the workplace, and exploring the close relationship between health and productivity. This intersection of distinct elements makes this field of study genuinely innovative and highly relevant.

Among the analysed articles, it was concluded that all of them found a positive relationship between exposure to nature (natural or simulated) and the proposed outcomes. However, it was observed, for example, that the studies did not assess the amount of time needed for nature exposure to actually influence the variables under study, making it difficult to understand whether the reported benefits are achieved with brief periods of exposure or whether a longer time is necessary for these effects to manifest significantly. This information is crucial for guiding interventions and practical strategies in the workplace. Only the studies by Yeom (2021) and Lei et al. (2021) focused on identifying the ideal amount of greenery. Determining the ideal amount of greenery is of utmost importance as it can provide crucial information for creating more effective restorative and biophilic work environments. If the amount of greenery is insufficient, restorative effects may be limited, and employees may not experience the expected benefits in terms of well-being, productivity, and job satisfaction. On the other hand, if there is an overabundance of natural elements (e.g., exceeding 20%, [49]), this may result in distractions or sensory overload, impairing performance and concentration in the work environment. Additionally, it was noted that studies concerning outdoor environments do not provide descriptions of whether there was or was not a sound evaluation; although, in an outdoor setting, we assume that surrounding sounds are present. However, it is not possible to say whether the positive results are solely due to the vision of green environments or are also related to hearing. Thus, it is noteworthy that vision and visual examples of nature dominate research in this area. Just like landscapes, spaces are also multisensory, and while there is evidence that natural sounds can lead to some form of restoration, the study of the impact of acoustic environmental factors should be reinforced in future investigations, like the analysis of visual properties of environments [61] or olfactory properties (e.g., [62]). By studying scents in work environments, for example, it is possible to identify biophilic design strategies that include the use of pleasant fragrances or the removal of undesirable odours. This can result in more welcoming, stimulating, and restorative environments for workers, improving the psychological environment and quality of work life. Also, few studies address the visual and auditory aspects of water as part of the restorative experience in the work environment, especially for urban workers living in waterfront areas. In this context, the possibility of visual contact with water is significant for these workers, and elements such as water colour and sound play an important role in restorative effects (e.g., [63,64]).

Although this review aimed to be exhaustive and comprehensive, there are some limitations to consider. For example, conducting a meta-analysis would have been relevant, but due to the lack of homogeneity found in study designs and the high variability of measures used in the selected articles, it was not possible to conduct one. There was a lack of standardization regarding the variables observed in the studies (e.g., physiological, psychological, and organizational). Moreover, even when the same construct was considered in various studies (e.g., job satisfaction), the measures used to assess it were different, introducing more variability in the findings and making generalization difficult. In this regard, and according to Moll et al. (2022), there is a need for standardization of the variables, tools, and experimental designs used in this type of research. Another limitation is related to potential double-counting, meaning that it was not controlled whether studies by the same authors used the same data set and reported the same correlations as previously published studies. This issue can affect the reliability of the results and should be considered in future studies to avoid overvaluing certain findings.

According to the previous literature reviews on the benefits of nature contact (e.g., [65]), our results show that exposure to nature offers a wide range of benefits for workers. However, notwithstanding the desire for biophilia, its fulfilment may not have the powerful effects often discussed. The extent and effects of employees’ contact with nature are still poorly understood in the work context [24]. This suggests that future studies should deepen the understanding of underlying mechanisms and explore the practical feasibility of implementing biophilic elements in work environments to achieve significant benefits. It can be concluded that workplaces should be designed to incorporate more green spaces, while seeking restorative interventions to assist in overcoming challenges related to productivity, performance, well-being, satisfaction, and employee health.

## 5. Conclusions

Although recovery mechanisms at work are mainly associated with activities outside of work, such as vacations, weekends, and breaks during the workday (e.g., [31,66,67]), there is a growing need to understand the role of restorative work environments in this recovery process. Restorative work environments are spaces designed to provide workers with a closer nature experience, with biophilic elements such as plants, water, natural light, and visual representations of nature. These natural elements in the workplace have been associated with benefits for well-being, mental health, productivity, and worker performance. Restorative work environments offer the possibility of promoting the recovery and restoration of workers’ internal resources within the work context itself. Thus, they become an extension of non-work activities, providing a continuous opportunity for restoration throughout the workday.

This systematic review provides a comprehensive overview of the literature results, pointing to both positive contributions and limitations as well as aspects that require more detailed future studies. By addressing these limitations and conducting more in-depth investigations, future studies have the potential to provide more evidence of, and valuable insights into, the restorative effects of nature in the work environment, benefiting both employees and organizations. It also provides a clearer understanding of the effects of exposure to nature in the workplace, bringing scientific evidence that can guide actions to promote healthier and more productive environments and contribute to more sustainable practices in the corporate world.

## Figures and Tables

**Table 1:** Inclusion and exclusion criteria used in the selection process.

Inclusion Criteria	Exclusion Criteria
Empirical studies	Study reviews
English language	No English language
Workers Characteristics of the nature (sound and green) of the workplace Effects on workers	Books and chapters Non-peer-reviewed articles Students Articles not complete or not accessible Related to non-work environments Telework

**Table 2:** Description of the 16 studies included in the systematic review.

	Ref.	Main Objective	Design	Sample Size	Type of Nature Exposure	Restorative Outcome	Downs and Black Score
1	[29]	- To illustrate the process of a research intervention aimed at designing a workplace, using a participatory design approach, and to consider the beneficial effect of restorative environments in reducing stressful elements and improving well-being at work.	Intervention research	57	Simulated nature (V/S)	Physical and Psychological Well-being Work engagement Job satisfaction	(21/26)
2	[49]	- To investigate the impacts of various greenery doses on workplace well-being from the perspectives of physiological, psychological, and productivity performance.	Experimental design	15	Simulated nature (V/S)	Physiological Psychological Productivity	(21/23)
3	[33]	- To examine whether a soundscape element perceived as pleasant has restorative effects in a simulated open-plan office	Experimental design	75	Simulated nature (V/S)	Physiological Psychological Task performance	(22/22)
4	[50]	- To evaluate the impact of a multisensory biophilic environment on occupants’ cognitive performance, stress, productivity, mood, connectedness to nature, and attention.	Experimental design	37	Simulated nature (V/S)	Physiological indicators of stress Attention restoration and fatigue Cognitive performance	(22/24)
5	[51]	- To examine whether exposure to biophilic indoor environments helps people recover from stress and anxiety and how those effects differ among different types of biophilic elements.	Experimental design	100	Simulated nature (V/S)	Physiological Psychological—anxiety level	(24/27)
6	[52]	- To measure blood pressure, heart rate, heart rate variability, and skin conductance level and administer cognitive tests to measure reaction time and creativity, through three versions of biophilic design in simulated open and enclosed office spaces in virtual reality (VR).	Experimental design	30	Simulated nature (V)	Physiological indicators of stress reaction Cognitive function—creativity	(23/26)
7	[37]	- To measure the emotional impact, task performance, and task load of the subjects according to four virtual experiments (a non-green wall, a freestanding green wall, two freestanding green walls, and a full-sized green wall).	Experimental design	27	Simulated nature (V)	Task performance Task load Positive and Negative affect	(21/23)
8	[30]	- To analyse changes in workplace sitting time and self-reported habit strength concerning uninterrupted sitting and PA during work, when relocating from a traditional setting to “active” biophilic-designed surroundings. - To assess possible changes in work-associated factors such as satisfaction with the environment, work engagement, and work performance, among office staff.	Correlational	12	Simulated nature (V)	Satisfaction with office environment Work engagement Job performance	(22/26)
9	[53]	- To examine the impacts of biophilic design attributes in offices on workers’ health and well-being. - To develop a new post-occupancy evaluation (POE) questionnaire for evaluating the biophilic design for workplace health and well-being.	Observational and correlational	201	Simulated nature (V)	Health and well-being	(19/24)
10	[54]	- To investigate whether access to a green outdoor environment at work is related to employees’ perceived level of stress and attitude toward the workplace.	Correlational	439	Access to a green outdoor environment at work (V/S)	Workplace greenery Level of stress Workplace attitude	(19/19)
11	[55]	- To investigate relations between various types of self-reported nature exposure at work and at home, and well-being among employees across two years.	Correlational	664	Window view Frequency of looking out of the window at work and at home Frequency of physical activities in natural surroundings (V/S)	Well-being (happiness, vitality, vigour, creativity)	(21/23)
12	[56]	- To investigate two-directional relations between various types of exposure to the natural world, at work and at home, and employee well-being.	Correlational	841	Number of indoor plants Type of view from the window Frequency of looking out of the window (V)	Well-being (happiness, vitality, vigour, creativity)	(21/23)
13	[57]	- To investigate how and why the workplace outdoor environment is used by office workers and the impact of these environments on office workers’ health and well-being.	Case study	402	- Use of outdoor environment during the workday - Activities performed in the outdoor environment at the workplace (V/S)	Health Job Satisfaction Workability	(18/22)
14	[58]	- To investigate the effects of natural elements and direct and indirect sunlight exposure on employees’ mental health and work attitudes.	Correlational	444	Natural elements exposure Sunlight exposure (V/S)	Depression Anxiety Job satisfaction Organizational commitment	(17/22)
15	[59]	- To examine the relationships between acute stress and alertness of nurses, and duration and content of exterior views from nurse work areas.	Correlational	32	Window view (V)	Chronic stress Acute stress Arousal	(17/22)
16	[60]	- To investigate the effect of window views on job satisfaction and stress.	Correlational	931	Window view (V)	Job stress Job satisfaction	(18/23)

(V)—visual stimuli; (S)—sound stimuli.

## Data Availability

No new data were created or analysed in this review. Data sharing does not apply to this article.
